# Non-negative matrix factorisation methods for the spectral decomposition of MRS data from human brain tumours

**DOI:** 10.1186/1471-2105-13-38

**Published:** 2012-03-08

**Authors:** Sandra Ortega-Martorell, Paulo JG Lisboa, Alfredo Vellido, Margarida Julià-Sapé, Carles Arús

**Affiliations:** 1 Departament de Bioquímica i Biología Molecular, Universitat Autònoma de Barcelona (UAB), Cerdanyola del Vallès, Spain; 2 Centro de Investigación Biomédica en Red en Bioingeniería, Biomateriales y Nanomedicina (CIBER-BBN), Barcelona Spain; 3 Institut de Biotecnologia i de Biomedicina, Universitat Autònoma de Barcelona, Cerdanyola del Vallès, Spain; 4 Department of Mathematics and Statistics, Liverpool John Moores University (LJMU), Liverpool, UK; 5 Department of Computer Languages and Systems, Universitat Politècnica de Catalunya (UPC), Barcelona, Spain

## Abstract

**Background:**

*In-vivo *single voxel proton magnetic resonance spectroscopy (SV ^1^H-MRS), coupled with supervised pattern recognition (PR) methods, has been widely used in clinical studies of discrimination of brain tumour types and follow-up of patients bearing abnormal brain masses. SV ^1^H-MRS provides useful biochemical information about the metabolic state of tumours and can be performed at short (< 45 ms) or long (> 45 ms) echo time (TE), each with particular advantages. Short-TE spectra are more adequate for detecting lipids, while the long-TE provides a much flatter signal baseline in between peaks but also negative signals for metabolites such as lactate. Both, lipids and lactate, are respectively indicative of specific metabolic processes taking place. Ideally, the information provided by both TE should be of use for clinical purposes. In this study, we characterise the performance of a range of Non-negative Matrix Factorisation (NMF) methods in two respects: first, to derive sources correlated with the mean spectra of known tissue types (tumours and normal tissue); second, taking the best performing NMF method for source separation, we compare its accuracy for class assignment when using the mixing matrix directly as a basis for classification, as against using the method for dimensionality reduction (DR). For this, we used SV ^1^H-MRS data with positive and negative peaks, from a widely tested SV ^1^H-MRS human brain tumour database.

**Results:**

The results reported in this paper reveal the advantage of using a recently described variant of NMF, namely Convex-NMF, as an unsupervised method of source extraction from SV^1^H-MRS. Most of the sources extracted in our experiments closely correspond to the mean spectra of some of the analysed tumour types. This similarity allows accurate diagnostic predictions to be made both in fully unsupervised mode and using Convex-NMF as a DR step previous to standard supervised classification. The obtained results are comparable to, or more accurate than those obtained with supervised techniques.

**Conclusions:**

The unsupervised properties of Convex-NMF place this approach one step ahead of classical label-requiring supervised methods for the discrimination of brain tumour types, as it accounts for their increasingly recognised molecular subtype heterogeneity. The application of Convex-NMF in computer assisted decision support systems is expected to facilitate further improvements in the uptake of MRS-derived information by clinicians.

## Background

### Introduction

The clinical investigation of an abnormal mass in the brain frequently starts with its non-invasive characterisation (localisation, infiltration, etc.), normally with a magnetic resonance imaging (MRI) study. Magnetic resonance spectroscopy (MRS) is another MR technique that, unlike MRI, provides insight into the biochemistry of tissue through a discrete signal in the frequency domain (a spectrum) containing information about the relative abundance of several low molecular weight metabolites, lipids and macromolecules in the millimolar range of concentration.

This MR modality has been used in computer-based systems for diagnostic decision support [1], building on the increasing availability of data in electronic format [2,3]. However, for brain tumours and, more specifically, glial tumours, the computer-based discrimination of the grade or the specific subtype of tumour still leaves a "gray zone" of uncertainty between class labels [4-6]. Therefore, it would be desirable to define decision support systems that were able to provide accurate discrimination of tumour types from the spectra without prior information regarding tumour type and grade. From the PR viewpoint, this is an unsupervised modelling task.

The MRS data analysed in the current work are single-voxel. That is, for each patient we have a single spectrum corresponding to a small volume located within the tumour core. The aim of this study is to separate the constituent source signals on the assumption that they are mixed linearly in each single-voxel spectral measurement. This is because, even within a single voxel, an heterogeneous mix of tissue types may be expected. In this way, the main constituents of the voxel could be separately identified and quantified, providing, in turn, a quantification of class (tumour type or healthy tissue) membership for the sources of each single voxel spectrum, as an alternative to the class labelling of the spectrum as a whole.

Linear unsupervised feature extraction PR techniques are commonly used in neuro-oncology for data preprocessing and dimensionality reduction (DR) previous to the diagnostic classification of brain tumours. The usual choices are principal component analysis (PCA) [7-9] and independent component analysis (ICA) [10-12]. PCA has mostly been used within a DR framework, and the extracted features lack a direct interpretation. In a recent study [13], PCA was applied in an alternative manner to represent each tumour type through mean and variability spectra for ulterior classification using an LCModel [14]. ICA, instead, goes beyond DR to provide source extraction, by identifying the sources that add together to form the measured MRS signal. As stated in [10], though, in analysing these type of data, ICA will often yield components that "would correspond with identifying the independent degrees of freedom in MRS, not with individual metabolites, but with characteristic tissue generators", or, in other words, constituent tissues that are present in different proportions in each of the voxels where MRS is measured. There is no guarantee that these tissue generators will be tumour type-specific and, therefore, there is little *a priori *evidence to support that these sources will suffice to infer accurate tumour type predictions [11]. The alternative to feature extraction for DR is feature selection [15-17]. Here, the interpretability of the results fully depends on the correspondence between the selected features (MRS frequencies) and known metabolites.

In this study, we characterise the performance of a range of variants of an unsupervised method of the matrix factorisation family, namely Non-negative Matrix Factorisation (NMF, [18,19]), in two respects: first, to derive sources correlated with the mean spectra of known tissue types; second, taking the best performing NMF method for source separation, we compare its accuracy for class assignment when using the mixing matrix directly as a basis for classification, as against using the method for DR. This method is unsupervised in the sense that labelled cases are not required to create a model of the analysed MRS data (i.e., to find the MRS sources). Conceptually, it lies somewhere in between PCA and ICA. In the spectroscopy-related bioinformatics domain, standard NMF has previously been used for disease classification from infrared spectroscopy blood serum data [20] and the related nonnegative PCA (NPCA) technique has been applied to the classification of different tumours from mass spectroscopy serum proteomic data [21]. Also within the oncology field, NMF has recently been used for DR in large scale gene expression data [22] and for recovering constituent sources from MR chemical shift imaging (CSI) of the brain, in a variant called constrained NMF [23].

The results reported in the current paper reveal the advantage of using one of the recently described NMF variants, namely Convex-NMF [24], as an unsupervised method of source extraction from SV ^1^H-MRS. In contrast with ICA, most of the sources extracted by the proposed technique closely correspond to the mean spectra of some of the analysed tumour types. This similarity allows accurate diagnostic predictions to be made for each patient (that is, for each SV spectrum) both in fully unsupervised mode or using Convex-NMF as a DR step previous to standard supervised classification. These predictions are comparable to or more accurate than those obtained with supervised techniques.

The remaining of the paper is organised as follows. The *Materials *subsection describes the data used in this study, while the *Methods *subsection summarises existing approaches for the application of NMF, then discusses how they are used with MRS data, presents different model initialisation methods, and also explains how to use the obtained information to label cases and to reduce data dimensionality. The *Results *section compiles and presents all the experimental results, with the objective of assessing NMF in fully unsupervised mode, and to investigate the use of NMF as a DR method previous to standard supervised classification. These results are later discussed and some conclusions are drawn.

### Materials

The data analysed in this study were extracted from INTERPRET, an international multi-centre database [2] resulting from the INTERPRET European research project^a ^[8]. Class labelling was performed according to the World Health Organisation system for diagnosing brain tumours by histopathological analysis of a biopsy sample. These are single-voxel proton MRS (SV-^1^H-MRS) data acquired at 1.5T and at two different echo times (short, 20-32 ms (STE) and long, 135-144 ms (LTE)) from brain tumour patients and healthy controls (that is, two spectra, one at STE and another at LTE, are available for each individual).

The importance of using two different signal acquisition conditions (STE and LTE) lies in the different metabolites that are detectable at each of them. STE is more sensitive to those with short T2 (an MR relaxation time parameter) values (it is, for example, more adequate to detect mobile lipids) and, in addition, all signal peaks are positive. On the other hand, in LTE spectra we can find both positive and negative peaks, where the negative peak is due to the inverted Alanine or Lactate doublets. The analysed data set included, at LTE, 20 astrocytomas grade II (A2), 78 glioblastomas (GL), 31 metastases (ME), 55 low-grade meningiomas (MM) and 15 normal brain parenchyma measurements from healthy controls (NO); at STE, it included 22 A2, 86 GL, 38 ME, 58 MM, and 22 NO. Data were processed as in [1]. A total of 195 clinically-relevant frequency intensity values measured in parts per million (ppm) were sampled from each spectrum in the [4.24,0.50] ppm interval. Unit length normalisation (UL2) of the spectra was performed.

A further test data set (not used for source extraction, but only for the validation of the obtained results) was gathered from three medical centres: Centre Diagnòstic Pedralbes (CDP), Institut d'Alta Tecnologia (IAT) and Institut de Diagnòstic per la Imatge (IDI)-Badalona in Barcelona, Spain. It was processed in the same conditions as the rest of the data, and consists of STE and LTE spectra from 56 patients and healthy controls: 10 A2, 40 high-grade aggressive tumours (30 GL + 10 ME), 3 MM, and 3 NO subjects.

MRS data were acquired according to the medical ethics regulations of the countries of the medical centres involved, in particular, with the Helsinki Declaration and the Spanish "*Ley Orgánica de Protección de Datos de Carácter Personal"(LOPD), Ley Orgánica 15/1999" *and the *"95/46/EU directive on data protection, December 13^th^, 1999"*. All patients or their legal representatives signed informed consent forms, agreeing to the study and to the use of their deidentified (anonymised) data for research.

## Methods

As stated in the introduction, NMF can be seen as a DR technique, functionally similar to source extraction. This section summarily describes some of the existing NMF methods and the different alternatives for their initialisation. The choice of initialisation technique turns out to be a key feature for the success of NMF as a tumour type classification method. The specific way in which these techniques are used and interpreted in the context of MRS data analysis is also described in this section. We later explain how the data can be labelled *a posteriori*, once the sources have been extracted, with the purpose of helping us to understand the extent to which obtained sources are able to represent the data. Finally, the way sources can be used strictly for DR is also described.

### Non-negative matrix factorisation methods for source extraction

In the standard NMF description [18], a non-negative matrix *V *of observed data (*d *× *n*, where *d *is the data dimensionality and *n *is the number of observations), is approximately factorised into two non-negative matrices, *W *(of dimensions *d *× *k*, where *k *is the number of data basis or sources, and *k *<*d*) and *H *(of dimensions *k *× *n*, each of whose columns provides the encoding of a data point: a SV spectrum in this study). The product of these two matrices provides a good approximation to the original matrix, that is, *V≈WH*. The conventional approach to finding the two factors is by minimising the divergence between *V *and *WH*:

$$\underset{W,H}{min}\;f(W,H)=\frac{1}{2}{\left\|V-W\;H\right\|}_{F}^{2}$$

subject to the non-negativity constraints mentioned above, where ||·||_*F *_is the Frobenius norm. In this study, the following divergence minimisation methods, which cover a wide palette of algorithmic alternatives, were considered:

• Euclidean distance update equations (herein referred to as *euc*) [19]

The objective function is optimised with multiplicative update rules for *W *and *H*:

$$W\leftarrow W\frac{VH^{T}}{WHH^{T}};\;\;\text{and}\;\;H\leftarrow H\frac{W^{T}V}{W^{T}WH}$$

Monotonic convergence of the algorithm can be proven [19]. These update equations preserve the nonnegativity of *W *and *H*, and constrain the columns of *W *to sum to unity.

• Alternating least squares (*als*) [18]

This technique alternately fixes one matrix and improves the other.

W←arg minW≥0f(W,H);  H←arg minH≥0f(W,H)

where *W *and *H *are updated as follows:

$$W\leftarrow {\left({\left(HH^{T}\right)}^{-1}HV^{T}\right)}^{T};\;\;\text{and}\;\;H\leftarrow {\left(W^{T}W\right)}^{-1}W^{T}V$$

setting all negative elements in *W *and *H *to zero.

• Alternating non-negative least squares using projected gradients (*alspg*) [25]

The equations for *W *and *H *in the alternating least squares method above are solved here using projected gradients. For *H*, this entails:

$$H\leftarrow P\left[H-\alpha \nabla \bar{f}\left(H\right)\right]$$

where α is the step size, and *P*[·] is a bounding function that ensures that the solution remains within the boundaries of feasibility. The gradient function is solved as:

$$\nabla \bar{f}\left(H\right)=W^{T}\left(WH-V\right)$$

The same approach is used to calculate *W*.

• Alternating least squares with Optimal Brain Surgeon (OBS) [26] (*alsobs*) [27,28] Similar to alternating least squares, this algorithm alternately solves the least squares equations for *W *and *H*. The negative elements in *W *and *H *are set to zero and the rest are adjusted using the OBSmethod, through second-order derivatives. The update rules for *W *and *H *are:

$$W\leftarrow {\left({\left(HH^{T}\right)}^{-1}HV^{T}\right)}^{T}+\delta_{W};\;\;\text{and}\;\;H\leftarrow {\left(W^{T}W\right)}^{-1}W^{T}V+\delta_{H}$$

where, δ_*W *_and δ_*H *_act as regularisation terms and are responsible for eliminating the less important elements of *W *and *H*, respectively (the original OBS was used as a weight pruning mechanism in artificial neural networks), thus re-adjusting the remaining elements optimally. More implementation details can be found in [28].

• Convex-NMF (*convex*) [24]. To achieve interpretability, this method imposes a constraint that the vectors (columns) defining *W *must lie within the column space of *V*, i.e. *W *= *VA *(where *A *is an auxiliary adaptative weight matrix that fully determines *W*), so that *V≈VAH*. By restricting *W *to convex combinations of the columns of *V *we can, in fact, understand each of the basis or sources as weighted sums of data points. Unlike the previous ones, this NMF variant applies to both nonnegative and mixed-sign data matrices. The factors *H *and *A *are updated as follows:

$$H^{T}\leftarrow H^{T}\sqrt{\frac{{\left(V^{T}V\right)}^{+}A+H^{T}A^{T}{\left(V^{T}V\right)}^{-}A}{{\left(V^{T}V\right)}^{-}A+H^{T}A^{T}{\left(V^{T}V\right)}^{+}A}};\;\;A\leftarrow A\sqrt{\frac{{\left(V^{T}V\right)}^{+}H^{T}+{\left(V^{T}V\right)}^{-}AHH^{T}}{{\left(V^{T}V\right)}^{-}H^{T}+{\left(V^{T}V\right)}^{+}AHH^{T}}}$$

where (·)^+ ^is the positive part of the matrix, where all negative values become zeros; and (·)^- ^is the negative part of the matrix, where all positive values become zeros. All the algorithms, for all initialisations, were allowed to achieve convergence. Such convergence was qualified as the lack of variation in the reconstruction error, from one iteration to the next, over a common set small threshold of value 10^-5^.

### Interpretation of the methods

In NMF for the analysis of MRS data, the rows in *H *can be understood as estimates of the concentration/abundance of the constituent signals or sources, while the columns in *W *are the corresponding constituent signals or sources of the spectra themselves. In conventional NMF methods (such as the first four previously described), the matrices *V, W *and *H *are constrained to be non-negative, thus permitting the interpretation of the mixing matrix entries as quantitative estimates of the amount of source tissue in the sample. The source can, as a result, be assigned to the class (tumour type or healthy tissue) with whose template it shows a higher correlation. If non-negativity is also imposed on the signals and sources, then it is commonplace truncating the negative values to zero, therefore loosing potentially relevant information (for instance, Lactate, Alanine, and Glutamine + Glutamate (Glx) in LTE spectra, which are expected to be especially relevant for discrimination between tumour types). Some of the methods described above impose the constraint of non-negativity only on the mixing elements representing the constituent tissue fractions. Where non-negative signals are also required, we propose using absolute values instead, in order to reduce data loss from the negative peaks.

Convex-NMF, instead, enforces this non-negative constraint only on *H*, while *V *and *W *are allowed to be of mixed sign. Given that the observed MRS data are of mixed sign, their sources should also be of mixed sign. Thus, understanding *W *as the source spectra matrix, the sources will be intuitively interpretable and no pre-processing of the spectra is required in order to make them non-negative, thus preventing any unnecessary loss of information (in the case of our database, losing the information in the negative peaks of the SV ^1^H-MRS LTE spectra). As in the previous methods, *H *can be understood as estimates of the concentration/abundance of the constituent signals.

### NMF initialisations

NMF methods unavoidably converge to local minima. As a result, the NMF bases will be different for different initialisations. In this study, six forms of initialisation were considered (with some variations depending on the method). Although a standard procedure to justify the choice of NMF initialisation does not exist, the six alternatives considered here cover a wide array of approaches: from random initialisation, to prototype-based clustering methods (K-means and Fuzzy C-Means, which provide a data density-based sample of initial data locations), and feature extraction techniques (PCA, ICA and NMF itself, which initialise the algorithm according to the basic eigenstructure of the data).

• Random:

[all methods]: *W *and *H *are initialised as dense matrices of random values between 0 and 1.

• K-means clustering:

[*euc, alspg, als, alsobs*]: *W *is initialised with the cluster centroids, and *H *with the distances from each point (MR spectrum) to every centroid.

[*convex*]: *H *is initialised as *H*^(0) ^*= C + *0.2*E*, where *E *is a matrix with all its elements equal to one, and *C *= (*c*_1_, . . ., *c_n_*) is filled with the cluster indicators, which are based on the cluster indices of each point, such that *C_ik _*= {0,1} and the ones indicate cluster membership. *A *is initialised as *A*^(0) ^= (*C *+ 0.2*E*)*D*^-1^, where *D *is a diagonal matrix with each element being the number of points in each cluster [24].

• Fuzzy C-Means (FCM):

[*euc, alspg, als, alsobs*]: *W *is initialised with the cluster centres, and *H *with the fuzzy partition matrix (or membership function matrix); as in [29].

[*convex*]: *H *is initialised as *H*^(0) ^= *C + *0.2*E*, where *C *here is filled with the fuzzy partition values, and *E *is a matrix with all its elements equal to one. *A *is initialised as *A*^(0) ^= (*C *+ 0.2*E*)*D*^-1 ^, where *D *is a diagonal matrix with each element being the number of points in each cluster.

• PCA:

[*euc, alspg, als, alsobs*]: The mean vector is subtracted from the complete dataset, and this is followed by the computation of its eigenvectors and eigenvalues. The matrix *W *is initialised with the whitened data (the corresponding projection of the eigenvectors), and *H *with the de-whitening matrix. In order to use the initial *W *and *H *matrices obtained from PCA in NMF, the negative values are truncated, as proposed in [29].

[*convex*]: *H *is initialised as *H*^(0) ^= *C *+ 0.2*E*, where *C *is the de-whitening matrix, calculated, as in the rest of methods, after calculating PCA, and also truncating the negative values. For the initialisation of *A*, and as suggested in [24], first we compute *A *= *H^T^*(*HH^T^*)^-1^, and then *A*^(0) ^= (*A*)^+ ^+ 0.2*E*〈(*A*)^+^〉 so that the negative elements are removed, where 〈*X*〉 = ∑_*n, k*_|*X_n, k_*|/||*X_n, k_*||_0_, and where ||*X_n, k_*||_0 _is the number of nonzero elements in *X*.

• ICA (FastICA [30] algorithm):

[*euc, alspg, als, alsobs*]: The independent components extracted using FastICA are used to initialise *W*, and *H *is initialised with the resulting mixing matrix. Then, to meet the non-negativity condition of NMF, the negative values are truncated.

[*convex*]: *H *and *A *are initialised as in the PCA (for *convex*) initialisation, with the only difference that *H *is filled with the sources or independent components from FastICA.

• Non-negative Matrix Factorisation (NMF, *als *algorithm):

[*euc, alspg, als, alsobs*]: *W *is initialised with the sources extracted with NMF (*als*), and *H *is initialised with the resulting mixing matrix. In the case of *als *method, initialising with the same method is equivalent to duplicating the number of iterations, which does not necessarily mean that the results will improve.

[*convex*]: *H *and *A *are initialised as in the PCA and FastICA (for *convex*) initialisations, with the only difference that *H *is filled with the sources from NMF (*als*).

In principle, we might expect the different initialisation strategies to behave as follows. Random initialisation might be considered as an uninformed first estimate for NMF methods, which may lead to different outcomes given different initialisation conditions [29,31]. We might expect K-means and FCM initialisations to make all methods perform better, but the results may depend on the initial selection of clusters; therefore, different results could be obtained depending on such selection. PCA and ICA, instead, can provide a unique solution, although perhaps too biased, while, in the case of NMF, the existence of a unique solution will depend on its own initialisation. All these methods will converge to local optima, so there is no guarantee that the solution obtained will be the best possible.

### Tumour type labelling using the mixing matrix and the sources

As explained in the introduction section, NMF is used in this study as an unsupervised method in the sense that labelled MRS cases are not used to create the data model. The obvious advantage of this approach is that the labelling procedure can be made independent of any specific labelled (or mislabelled) MRS dataset that might bias the generalisation capabilities of the model.

In order to determine how well the sources obtained through NMF represent the data, we propose to infer the labels of the data only on the basis of the mixing matrix and the source signals calculated, which will give us an idea of the extent to which the sources contribute to the reconstruction of each MRS observation (or patient case). The calculation of the contribution *C *of each source *k *to each case *i *is:

$$C_{\left(i,k\right)}=V_{i}^{T}W_{k}^{}H_{\left(k,i\right)}$$

where *V *the data matrix, *W *is the matrix of sources, and *H *is the mixing matrix. The predicted label can then be inferred from the values in *C *as follows: for each case *i*, the label is provided by the source *k *that has the highest value of contribution for that case.

### Source extraction as a dimensionality reduction procedure prior to classification

The description of the MR spectra through a limited number of extracted sources also entails a DR process in the form of feature extraction. As previously mentioned, the use of DR methods in the form of feature selection or extraction is commonplace in the analysis of MRS. The extracted features can then be used for traditional classification, within a standard supervised framework using labelled cases. This was accomplished in the current study using the Gram-Schmidt process [32] for orthonormalising the set of obtained source signals. This method takes a finite, linearly independent set *W = W_1_*, . . ., *W_k _*for *k *≤ *n*, where *k *is the number of sources and *n *is the number of samples, and generates an orthogonal set *W*' = *U*_1_, ..., *U_k _*that spans the same *k*-dimensional subspace of ℜ^*n *^as *W*.

## Results

In this section, we compile and present all the experimental results. The objective of the experiments was twofold: first, the assessment of NMF in fully unsupervised mode as a source extraction and tumour type-labelling method and, second, the evaluation of NMF as a DR method prior to standard supervised classification.

### NMF as a source extraction method

Here, we provide the comparative results of the application of the five NMF methods for source extraction outlined in the *Methods *section, for each of the six different initialisation strategies discussed. The goal was to find the best combination of NMF method and initialisation for the type of data analysed. Experiments were carried out for four different brain tumour diagnostic problems from MRS acquired both at LTE and STE. In each of these classification problems, we attempted to discriminate between one or two tumour types and healthy tissue, namely A2 *vs*. NO; A2 *vs*. ME *vs*. NO; A2 *vs*. GL *vs*. NO; and A2 *vs*. MM *vs*. NO.

A2 are low-grade (grade II on a scale I-IV of the WHO [33]) glial tumours with an infiltrating behaviour (they grow by infiltrating normal brain tissue). They evolve (directly or through an intermediate anaplastic glioma stage, WHO grade III) to GL, which are highly malignant, WHO grade IV tumours. ME are also grade IV tumours, but they have a different origin: They are tumours originated at other parts of the body that spread (they become metastatic) to distant sites, such as the brain. Grade IV tumours usually have a necrotic pattern, with strong lipid signals that are most evident when obtaining MRS data at short times of echo [34]. However, not all GL have this necrotic pattern, and some retain a spectral pattern which is overall similar to that of their low-grade glial counterparts, the A2, and might be considered as atypical within their type, or class outliers [35,36]. MM are low grade tumours (WHO grade I), from a completely different origin: meningeal cells. They have a distinct spectral pattern at LTE, with an inverted alanine doublet at ca. 1.45 ppm [8]. Their spectral pattern is also easy to recognise at STE, without necrosis, and it is different from the glial, metastatic, or normal patterns.

In summary, the choice of these specific problems at both time of echo acquisition conditions ultimately aimed to find answers to the following questions: 1) (A2 *vs*. NO): Is normal brain correctly distinguished from infiltrative tumour? 2) (A2 *vs*. ME *vs*. NO): Are grades (II *vs*. IV) well differentiated and distinct from normal tissue? 3) (A2 *vs*. GL *vs*. NO): Are grades still well recognised when one of the classes is heterogeneous? 4) (A2 *vs*.MM *vs*. NO): Can low grades (A2 *vs*. MM, or grade II *vs*. I), or infiltrative *vs*. non-infiltrative be differentiated?

Tables 1 and 2 compile the results of the correlation between the mean spectrum of each class (tumour type or healthy tissue from controls) and the source signal, extracted with NMF, that best represents this class, i.e. the source signal that has the highest correlation with the class. The number of sources calculated was selected according to the number of classes involved in each diagnostic problem studied. Calculating the correlation provides us with an indicator of to what extent each source is tumour-type specific.

**Table 1:** Summary of the results for LTE.

Experiment: LTE. A2, NO. (2 sources)						
	Random	K-means	FCM	PCA	FastICA	NMF (als)

euc	A2: 0.98	A2: 0.97	A2: 0.98	A2: 0.91	A2: 0.98	A2: 0.97
	NO: 0.96	NO: 0.99	NO: 0.99	NO: 0.98	NO: 0.87	NO: 0.95

als	A2: 0.97	A2: 0.97	A2: 0.97	A2: 0.97	A2: 0.97	A2: 0.97
	NO: 0.98	NO: 1.00	NO: 1.00	NO: 1.00	NO: 0.95	NO: 0.96

alspg	A2: 0.97	A2: 0.97	A2: 0.97	A2: 0.97	A2: 0.97	A2: 0.97
	NO: 0.96	NO: 1.00	NO: 1.00	NO: 1.00	NO: 0.95	NO: 0.96

alsobs	A2: 0.97	A2: 0.97	A2: 0.97	A2: 0.97	A2: 0.97	A2: 0.97
	NO: 0.96	NO: 1.00	NO: 1.00	NO: 1.00	NO: 0.95	NO: 0.99

convex	A2: 1.00	A2: 1.00	A2: 1.00	A2: 0.99	A2: 1.00	A2: 1.00
	NO: 1.00	NO: 1.00	NO: 1.00	NO: 1.00	NO: 1.00	NO: 1.00

**Experiment: LTE. A2, ME, NO. (3 sources)**						

	Random	K-means	FCM	PCA	FastICA	NMF (als)

euc	A2: 0.94	A2: 0.94	A2: 0.94	A2: 0.88	A2: 0.94	A2: 0.94
	ME: 0.85	ME: 0.85	ME: 0.85	ME: 0.78	ME: 0.86	ME: 0.85
	NO: 0.95	NO: 1.00	NO: 0.99	NO: 0.90	NO: 0.92	NO: 0.93

als	A2: 0.94	A2: 0.94	A2: 0.94	A2: 0.94	A2: 0.94	A2: 0.94
	ME: 0.85	ME: 0.84	ME: 0.84	ME: 0.85	ME: 0.85	ME: 0.85
	NO: 0.92	NO: 0.99	NO: 0.99	NO: 0.92	NO: 0.91	NO: 0.91

alspg	A2: 0.94	A2: 0.94	A2: 0.94	A2: 0.94	A2: 0.94	A2: 0.94
	ME: 0.85	ME: 0.85	ME: 0.85	ME: 0.85	ME: 0.85	ME: 0.85
	NO: 0.99	NO: 0.99	NO: 0.99	NO: 0.95	NO: 0.93	NO: 0.92

alsobs	A2: 0.94	A2: 0.94	A2: 0.94	A2: 0.94	A2: 0.94	A2: 0.94
	ME: 0.85	ME: 0.85	ME: 0.85	ME: 0.85	ME: 0.85	ME: 0.85
	NO: 0.96	NO: 0.99	NO: 0.99	NO: 0.95	NO: 0.95	NO: 0.95

convex	A2: 0.99	A2: 0.99	A2: 0.98	A2: 0.98	A2: 0.98	A2: 0.98
	ME: 0.88	ME: 0.88	ME: 0.90	ME: 0.86	ME: 0.87	ME: 0.87
	NO: 1.00	NO: 1.00	NO: 1.00	NO: 1.00	NO: 1.00	NO: 1.00

**Experiment: LTE. A2, GL, NO. (3 sources)**						

	Random	K-means	FCM	PCA	FastICA	NMF (als)

euc	A2: 0.91	A2: 0.91	A2: 0.91	A2: 0.87	A2: 0.92	A2: 0.91
	GL: 0.75	GL: 0.76	GL: 0.75	GL: 0.92	GL: 0.78	GL: 0.76
	NO: 0.99	NO: 0.99	NO: 0.99	NO: 0.96	NO: 0.94	NO: 0.99

als	A2: 0.92	A2: 0.92	A2: 0.92	A2: 0.92	A2: 0.92	A2: 0.92
	GL: 0.76	GL: 0.76	GL: 0.76	GL: 0.76	GL: 0.76	GL: 0.76
	NO: 0.95	NO: 0.99	NO: 0.99	NO: 0.99	NO: 0.95	NO: 0.98

alspg	A2: 0.91	A2: 0.91	A2: 0.91	A2: 0.91	A2: 0.91	A2: 0.91
	GL: 0.75	GL: 0.75	GL: 0.75	GL: 0.75	GL: 0.75	GL: 0.75
	NO: 0.99	NO: 0.99	NO: 0.99	NO: 0.99	NO: 0.97	NO: 0.96

alsobs	A2: 0.91	A2: 0.91	A2: 0.91	A2: 0.91	A2: 0.91	A2: 0.91
	GL: 0.75	GL: 0.75	GL: 0.75	GL: 0.75	GL: 0.75	GL: 0.75
	NO: 0.99	NO: 0.99	NO: 0.99	NO: 0.99	NO: 0.98	NO: 0.99

convex	A2: 0.94	A2: 0.97	A2: 0.95	A2: 0.94	A2: 0.96	A2: 0.96
	GL: 0.80	GL: 0.73	GL: 0.77	GL: 0.81	GL: 0.75	GL: 0.74
	NO: 0.98	NO: 1.00	NO: 1.00	NO: 0.99	NO: 1.00	NO: 1.00

**Experiment: LTE. A2, MM, NO. (3 sources)**						

	Random	K-means	FCM	PCA	FastICA	NMF (als)

euc	A2: 0.96	A2: 0.88	A2: 0.89	A2: 0.97	A2: 0.88	A2: 0.88
	MM: 0.92	MM: 0.97	MM: 0.97	MM: 0.91	MM: 0.99	MM: 0.97
	NO: 0.95	NO: 0.98	NO: 0.98	NO: 0.72	NO: 0.89	NO: 0.98

als	A2: 0.88	A2: 0.88	A2: 0.88	A2: 0.88	A2: 0.88	A2: 0.88
	MM: 0.97	MM: 0.97	MM: 0.97	MM: 0.97	MM: 0.97	MM: 0.97
	NO: 0.98	NO: 0.97	NO: 0.97	NO: 0.98	NO: 0.97	NO: 0.98

alspg	A2: 0.88	A2: 0.88	A2: 0.88	A2: 0.88	A2: 0.88	A2: 0.88
	MM: 0.97	MM: 0.97	MM: 0.97	MM: 0.97	MM: 0.97	MM: 0.97
	NO: 0.97	NO: 0.98	NO: 0.98	NO: 0.98	NO: 0.96	NO: 0.98

alsobs	A2: 0.88	A2: 0.88	A2: 0.88	A2: 0.88	A2: 0.88	A2: 0.88
	MM: 0.98	MM: 0.98	MM: 0.97	MM: 0.97	MM: 0.97	MM: 0.98
	NO: 0.98	NO: 0.98	NO: 0.98	NO: 0.98	NO: 0.97	NO: 0.98

convex	A2: 0.98	A2: 0.99	A2: 0.98	A2: 0.98	A2: 0.99	A2: 0.88
	MM: 1.00	MM: 0.99	MM: 0.99	MM: 0.99	MM: 1.00	MM: 0.98
	NO: 1.00	NO: 1.00	NO: 1.00	NO: 1.00	NO: 1.00	NO: 0.99

Summary of the correlation values for the sources most highly correlating with each type of tissue as expressed by its mean spectrum, for different diagnostic problems at LTE, and for all the NMF methods and initialisation conditions in the study. The diagnostic problems are: A2, NO; A2, ME, NO; A2, GL, NO; and A2, MM, NO

**Table 2:** Summary of the results for STE.

Experiment: STE. A2, NO. (2 sources)						
	Random	K-means	FCM	PCA	FastICA	NMF (als)

euc	A2: 0.94	A2: 0.94	A2: 0.94	A2: .83	A2: 0.96	A2: 0.92
	NO: 0.87	NO: 0.97	NO: 0.96	NO: 0.93	NO: 0.76	NO: 0.75

als	A2: 0.92	A2: 0.95	A2: .95	A2: .92	A2: 0.93	A2: 0.92
	NO: 0.75	NO: 0.96	NO: 0.96	NO: 0.96	NO: 0.83	NO: 0.75

alspg	A2: 0.93	A2: 0.95	A2: .95	A2: .92	A2: 0.95	A2: 0.92
	NO: 0.75	NO: 0.96	NO: 0.96	NO: 0.96	NO: 0.75	NO: 0.75

alsobs	A2: 0.92	A2: 0.94	A2: .94	A2: .92	A2: 0.95	A2: 0.92
	NO: 0.76	NO: 0.96	NO: 0.96	NO: 0.96	NO: 0.75	NO: 0.75

convex	A2: 0.99	A2: 0.99	A2: .99	A2: .98	A2: 0.99	A2: .99
	NO: 0.99	NO: 1.00	NO: 1.00	NO: 1.00	NO: 0.99	NO: 1.00

**Experiment: STE. A2, ME, NO. (3 sources)**						

	Random	K-means	FCM	PCA	FastICA	NMF (als)

euc	A2: 0.93	A2: 0.94	A2: 0.93	A2: 0.91	A2: 0.94	A2: 0.93
	ME: 0.98	ME: 0.98	ME: 0.98	ME: 0.86	ME: 0.99	ME: 0.98
	NO: 0.83	NO: 0.80	NO: 0.89	NO: 0.87	NO: 0.86	NO: 0.74

als	A2: 0.93	A2: 0.94	A2: 0.94	A2: 0.94	A2: 0.94	A2: 0.93
	ME: 0.98	ME: 0.98	ME: 0.98	ME: 0.98	ME: 0.98	ME: 0.98
	NO: 0.75	NO: 0.74	NO: 0.74	NO: 0.74	NO: 0.74	NO: 0.74

alspg	A2: 0.94	A2: 0.93	A2: 0.93	A2: 0.94	A2: 0.94	A2: 0.94
	ME: 0.98	ME: 0.98	ME: 0.98	ME: 0.98	ME: 0.98	ME: 0.98
	NO: 0.69	NO: 0.73	NO: 0.73	NO: 0.69	NO: 0.70	NO: 0.74

alsobs	A2: 0.94	A2: 0.94	A2: 0.94	A2: 0.94	A2: 0.94	A2: 0.94
	ME: 0.98	ME: 0.98	ME: 0.98	ME: 0.98	ME: 0.98	ME: 0.98
	NO: 0.69	NO: 0.69	NO: 0.72	NO: 0.69	NO: 0.71	NO: 0.69

convex	A2: 0.98	A2: 0.99	A2: 0.99	A2: 0.91	A2: 0.99	A2: 0.99
	ME: 1.00	ME: 1.00	ME: 1.00	ME: 0.99	ME: 0.99	ME: 0.99
	NO: 0.99	NO: 1.00	NO: 1.00	NO: 0.93	NO: 0.99	NO: 0.99

**Experiment: STE. A2, GL, NO. (3 sources)**						

	Random	K-means	FCM	PCA	FastICA	NMF (als)

euc	A2: 0.94	A2: 0.91	A2: 0.91	A2: 0.70	A2: 0.95	A2: 0.91
	GL: 0.95	GL: 0.91	GL: 0.94	GL: 0.56	GL: 0.96	GL: 0.95
	NO: 0.81	NO: 0.92	NO: 0.92	NO: 0.65	NO: 0.85	NO: 0.80

als	A2: 0.91	A2: 0.90	A2: 0.90	A2: 0.90	A2: 0.91	A2: 0.90
	GL: 0.95	GL: 0.95	GL: 0.95	GL: 0.95	GL: 0.95	GL: 0.95
	NO: 0.76	NO: 0.90	NO: 0.89	NO: 0.83	NO: 0.79	NO: 0.82

alspg	A2: 0.90	A2: 0.91	A2: 0.91	A2: 0.90	A2: 0.93	A2: 0.90
	GL: 0.95	GL: 0.93	GL: 0.93	GL: 0.95	GL: 0.95	GL: 0.95
	NO: 0.84	NO: 0.93	NO: 0.93	NO: 0.85	NO: 0.72	NO: 0.83

alsobs	A2: 0.93	A2: 0.91	A2: 0.91	A2: 0.91	A2: 0.92	A2: 0.92
	GL: 0.95	GL: 0.95	GL: 0.93	GL: 0.95	GL: 0.95	GL: 0.95
	NO: 0.72	NO: 0.80	NO: 0.93	NO: 0.80	NO: 0.72	NO: 0.74

convex	A2: 0.95	A2: 0.98	A2: 0.94	A2: 0.94	A2: 0.99	A2: 0.99
	GL: 0.98	GL: 0.98	GL: 0.98	GL: 0.98	GL: 0.98	GL: 0.98
	NO: 0.94	NO: 1.00	NO: 0.94	NO: 0.95	NO: 0.99	NO: 1.00

**Experiment: STE. A2, MM, NO. (3 sources)**						

	Random	K-means	FCM	PCA	FastICA	NMF (als)

euc	A2: 0.93	A2: 0.91	A2: 0.95	A2: 0.83	A2: 0.94	A2: 0.92
	MM: 0.57	MM: 0.56	MM: 0.54	MM: 0.74	MM: 0.64	MM: 0.57
	NO: 0.83	NO: 0.95	NO: 0.73	NO: 0.89	NO: 0.77	NO: 0.79

als	A2: 0.93	A2: 0.93	A2: 0.93	A2: 0.92	A2: 0.93	A2: 0.92
	MM: 0.57	MM: 0.57	MM: 0.57	MM: 0.57	MM: 0.57	MM: 0.57
	NO: 0.76	NO: 0.79	NO: 0.77	NO: 0.75	NO: 0.76	NO: 0.75

alspg	A2: 0.92	A2: 0.93	A2: 0.91	A2: 0.91	A2: 0.92	A2: 0.92
	MM: 0.57	MM: 0.57	MM: 0.57	MM: 0.57	MM: 0.57	MM: 0.57
	NO: 0.77	NO: 0.80	NO: 0.81	NO: 0.81	NO: 0.80	NO: 0.75

alsobs	A2: 0.92	A2: 0.92	A2: 0.92	A2: 0.92	A2: 0.92	A2: 0.92
	MM: 0.57	MM: 0.57	MM: 0.57	MM: 0.57	MM: 0.57	MM: 0.57
	NO: 0.77	NO: 0.80	NO: 0.80	NO: 0.80	NO: 0.76	NO: 0.77

convex	A2: 0.95	A2: 0.98	A2: 0.93	A2: 0.96	A2: 0.98	A2: 0.98
	MM: 0.98	MM: 0.90	MM: 0.91	MM: 0.79	MM: 0.86	MM: 0.85
	NO: 0.91	NO: 1.00	NO: 0.95	NO: 0.98	NO: 0.99	NO: 0.98

Summary of the correlation values for the sources most highly correlating with each type of tissue as expressed by its mean spectrum, for different diagnostic problems at STE, and for all the NMF methods and the initialisation conditions in the study. The diagnostic problems are: A2, NO; A2, ME, NO; A2, GL, NO; and A2, MM, NO

**Figure 1:**
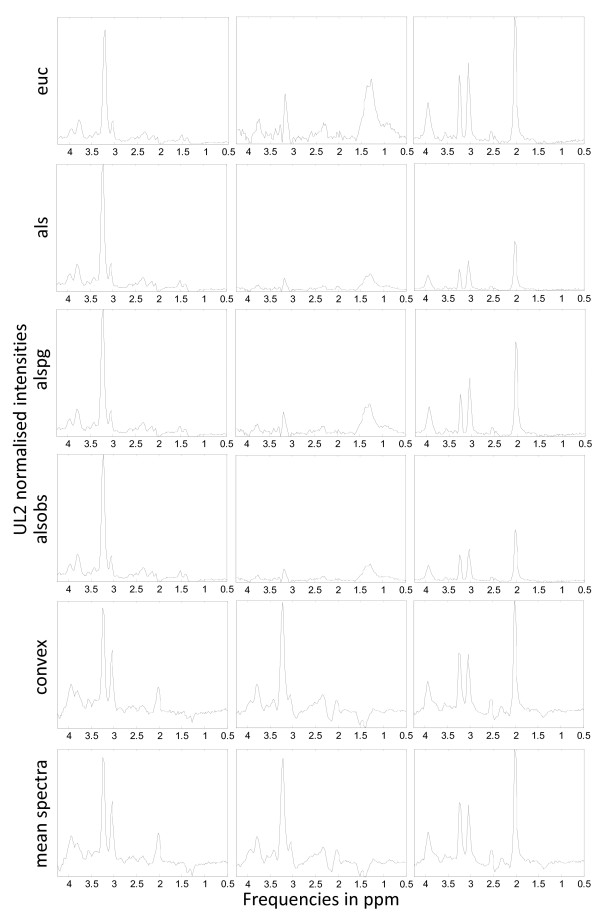
**Sources extracted in the experiment A2, MM, NO at LTE**. The first five rows show the source signals obtained in the experiments with A2, MM and NO at LTE, for all the methods under study and K-means clustering initialisation. The last row shows, from left to right, the mean spectra of A2, MM and NO, at LTE. Horizontal axis, for all plots: frequency in ppm scale. Vertical axis, for all plots: UL2 normalised intensity. The range of the vertical scales is fixed for each experiment and they are the same as those of the mean spectra of the last row, for comparative purposes.

Figure 1 is a graphical illustrative example of the obtained sources in the experiment A2 *vs*. MM *vs*. NO at LTE, for all the methods, with the K-means initialisation. The last row of the figure shows the mean spectra of the classes involved in this experiment, to be used as reference.

The computation times for the different methods used in this study, in a personal computer (memory (RAM): 4 GB, processor: Pentium Dual-Core T4400, 64-bit operating system), were less than one second in almost all cases, with the exception of *alsobs *(*euc*: 0.2, *als*: 0.4, *alspg*: 0.9, *alsobs*: 2.9, and *convex*: 0.8). The different initialisations added less than one second to the total computation time.

### Labelling using convex-NMF

The results summarised in Tables 1 and 2 lead to one clear conclusion: Convex-NMF was, consistently, the variant of NMF that yielded the highest correlations between the mean spectrum of the tumour types and the corresponding extracted sources. Convex-NMF was, therefore, the method of choice for the subsequent experiments.

We next report the results of the unsupervised labelling process: That is, the assignment of class labels (tumour types and healthy tissue) to each of the cases using the extracted sources and without modelling explicitly the relationship between the sources and the class labels. Table 3 shows the accuracy results (percentage of correct classification, total and by class) of the labelling process using Convex-NMF, for the same four diagnostic problems used to assess source extraction. To further assess the performance of Convex-NMF, we added here two more complex diagnostic problems with data acquired both at LTE and STE: the discrimination between A2, GL+ME (a superclass of the aggressive grade IV tumours: AG), NO; and A2, AG, MM. These are both classical discrimination problems in brain tumour diagnosis using MRS [7,9,37,38]. These two specific problems aim to answer the question: Are grades well recognised when one of the classes (AG) is heterogeneous (i.e. spectral pattern sub-types)?

**Table 3:** Labelling accuracy results obtained using Convex-NMF.

LTE					
**A2, NO(2SS)**	**A2, ME, NO(3SS)**	**A2, GL, NO(3SS)**	**A2, MM, NO(3SS)**	**A2, AG, NO(4SS)**	**A2, AG, MM(4SS)**

Total:100%(35/35)	Total:84.8%(56/66)	Total:71.1%(81/113)	Total:96.7%(87/90)	Total:77.8%(112/144)	Total:73.9%(136/184)
A2:100%(20/20)	A2:100%(20/20)	A2:100%(20/20)	A2:100%(20/20)	A2:100%(20/20)	A2:95.0%(19/20)
NO:100%(15/15)	ME:67.7%(21/31)	GL:59.0%(46/78)	MM:94.5%(52/55)	AG:70.6%(77/109)	AG:64.2%(70/109)
	NO:100%(15/15)	NO:100%(15/15)	NO:100%(15/15)	NO:100%(15/15)	MM:85.5%(47/55)

**STE**					

**A2, NO(2SS)**	**A2, ME, NO(3SS)**	**A2, GL, NO(3SS)**	**A2, MM, NO(3SS)**	**A2, AG, NO(4SS)**	**A2, AG, MM(4SS)**

Total:93.2%(41/44)	Total:91.5%(75/82)	Total:88.5%(115/130)	Total:89.2%(91/102)	Total:92.9%(156/168)	Total:86.3%(176/204)
A2:86.4%(19/22)	A2:77.3%(17/22)	A2:81.8%(18/22)	A2:77.3%(17/22)	A2:81.8%(18/22)	A2:90.9%(20/22)
NO:100%(22/22)	NO:100%(22/22)	GL:87.2%(75/86)	MM:89.7%(52/58)	AG:93.5%(116/124)	AG:87.9%(109/124)
	ME:94.7%(36/38)	NO:100%(22/22)	NO:100%(22/22)	NO:100%(22/22)	MM:81.0%(47/58)

Summary of the labelling accuracy using Convex-NMF with K-means initialisation for A2, NO; A2, ME, NO; A2, GL, NO; A2, MM, NO; A2, AG, NO; and A2, AG, MM, at LTE and STE. They include the accuracy (total and by tumour type), and the number of correctly labelled samples from the total, in parentheses. The number of source signals (SS) used in the experiments is indicated in parentheses

**Figure 2:**
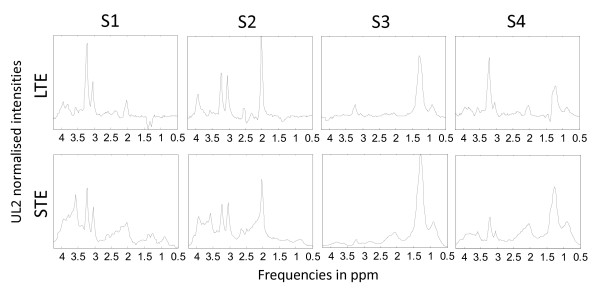
**Sources extracted in the experiment A2, AG, NO at LTE and STE**. Source signals obtained in the experiments with A2, AG (GL+ME) and NO at LTE (first row) and STE (second row), calculated with Convex-NMF, and initialised with K-means clustering. The sources in the first column (S1) represent A2, the ones in the second column (S2) represent NO, and the ones in the last two columns (S3 and S4) mainly represent AG. Axes labels and representation as in Figure 1.

**Figure 3:**
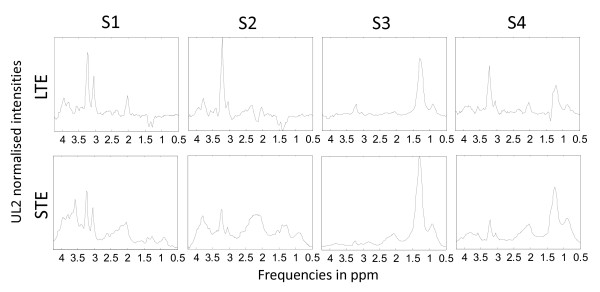
**Sources extracted in the experiment A2, AG, MM at LTE and STE**. Source signals obtained in the experiments with A2, AG (GL+ME) and MM at LTE (first row) and STE (second row), calculated with Convex-NMF, and initialised with K-means clustering. The sources in the first column (S1) again represent A2, the ones in the second column (S2) represent MM, and the ones in the last two columns (S3 and S4) again mainly represent AG. Axes labels and representation as in previous figures.

Convex-NMF was also initialised with K-means clustering, and a total of 4 source signals were calculated for these two problems, given that 4 classes were involved. The predicted labels were then used to determine to what extent each observation was correctly labelled, according to the INTERPRET database information. The results of the six diagnostic problems are compiled in Table 3, and Figures 2 and 3.

In the next section we use the sources in the context of supervised classification, and compare the results with equivalent classifiers, using the same settings.

### NMF for classification

#### Using convex-NMF extracted source signals for dimensionality reduction prior to classification

We now switch to experiments that analyse the use of Convex-NMF as a dimensionality reduction technique to preprocess the MRS data prior to standard classification. For this, we used the orthogonal set corresponding to the source signals obtained, and projected the data onto this basis. The SpectraClassifier^b ^software [39] was used to develop standard Fisher Linear Discriminant Analysis (LDA) classifiers, which were then evaluated through bootstrap with 1,000 repetitions. The results are shown in Table 4.

**Table 4:** Classification results using Convex-NMF for DR prior to classification with Fisher LDA.

LTE					
**A2, NO(2SS)**	**A2, ME, NO(3SS)**	**A2, GL, NO(3SS)**	**A2, MM, NO(3SS)**	**A2, AG, NO(4SS)**	**A2, AG, MM(4SS)**

Total:100% ± 0.0	Total:92.6% ± 3.3	Total:85.1% ± 3.4	Total:97.7% ± 1.6	Total:90.9% ± 2.4	Total:79.4% ± 3.0
A2:100% ± 0.0	A2:100% ± 0.0	A2:84.9% ± 8.5	A2:94.8% ± 5.0	A2:100% ± 0.0	A2:94.9% ± 5.2
NO:100% ± 0.0	ME:84.1% ± 6.8	GL:82.3% ± 4.3	GL:98.2% ± 1.9	AG:88.0% ± 3.1	AG:72.5% ± 4.3
	NO:100% ± 0.0	NO:100% ± 0.0	NO:100% ± 0.0	NO:100% ± 0.0	MM:87.5% ± 4.3

**STE**					

**A2, NO(2SS)**	**A2, ME, NO(3SS)**	**A2, GL, NO(3SS)**	**A2, MM, NO(3SS)**	**A2, AG, NO(4SS)**	**A2, AG, MM(4SS)**

Total:95.5% ± 3.1	Total:94.0% ± 2.6	Total:91.0% ± 2.5	Total:92.2% ± 2.7	Total:92.3% ± 2.0	TOTAL:87.7% ± 2.3
A2:91.2% ± 6.0	A2:86.3% ± 7.4	A2:82.5% ± 8.1	A2:86.3% ± 7.7	A2:81.9% ± 8.2	A2:95.5% ± 4.6
NO:100% ± 0.0	ME:95.0% ± 3.5	GL:90.9% ± 3.1	GL:91.5% ± 3.7	AG:92.8% ± 2.3	AG:86.3% ± 3.2
	NO:100% ± 0.0	NO:100% ± 0.0	NO:100% ± 0.0	NO:100% ± 0.0	MM:87.7% ± 4.3

Classification results (accuracy ± standard deviation) obtained with Fisher LDA (implemented in SpectraClassifier) for six diagnostic problems, from the source signals obtained by Convex-NMF, for data acquired at LTE and STE. Classifier results were validated through bootstrap. The number of source signals (SS) used in the experiments is indicated in parentheses

In order to compare these results with those of a traditional feature extraction method, we replicated all experiments using the SpectraClassifier software with PCA as data preprocessing feature extraction method (extracting a number of principal components equal to the number of source signals calculated for the corresponding NMF experiment). This was again followed by Fisher LDA classification and evaluated through bootstrap with 1,000 repetitions, as in the experiments in Table 4. The combination of PCA+LDA has been widely used to develop MRS classifiers [7-9,37]. The results for experiments with PCA are compiled in Table 5. Results obtained using an independent test set are shown in Table 6 for both FE methods: PCA and Convex-NMF.

**Table 5:** Classification results using PCA for DR prior to classification with Fisher LDA.

LTE					
**A2, NO(2PC)**	**A2, ME, NO(3PC)**	**A2, GL, NO(3PC)**	**A2, MM, NO(3PC)**	**A2, AG, NO(4PC)**	**A2, AG, MM(4PC)**

Total:100% ± 0.0	Total:93.8% ± 3.1	Total:82.1% ± 3.6	Total:95.4% ± 2.2	Total:84.6% ± 3.0	Total:80.2% ± 2.9
A2:100% ± 0.0	A2:100% ± 0.0	A2:100.0% ± 0.0	A2:95.0% ± 5.1	A2:100% ± 0.0	A2:100% ± 0.0
NO:100% ± 0.0	ME:86.9% ± 6.1	GL:75.4% ± 4.9	MM:94.4% ± 3.1	AG:79.7% ± 3.9	AG:75.9% ± 4.2
	NO:100% ± 0.0	NO:93.2% ± 6.8	NO:100% ± 0.0	NO:100% ± 0.0	MM:81.6% ± 5.2

**STE**					

**A2, NO(2PC)**	**A2, ME, NO(3PC)**	**A2, GL, NO(3PC)**	**A2, MM, NO(3PC)**	**A2, AG, NO(4PC)**	**A2, AG, MM(4PC)**

Total:93.2% ± 3.9	Total:90.2% ± 3.3	Total:84.4% ± 3.2	Total:88.1% ± 3.2	Total:86.2% ± 2.7	Total:81.3% ± 2.7
A2:86.4% ± 7.4	A2:86.2% ± 7.5	A2:81.4% ± 8.4	A2:81.6% ± 8.4	A2:72.5% ± 9.6	A2:90.7% ± 6.4
NO:100% ± 0.0	ME:91.9% ± 4.4	GL:84.6% ± 3.8	MM:87.8% ± 4.2	AG:89.5% ± 2.8	AG:80.6% ± 3.5
	NO:91.1% ± 6.0	NO:86.2% ± 7.5	NO:95.5% ± 4.5	NO:81.5% ± 8.6	MM:79.2% ± 5.3

Classification results (accuracy ± standard deviation) obtained with Fisher LDA (implemented in SpectraClassifier) for six diagnostic problems, from the source signals obtained by PCA, for data acquired at LTE and STE. Classifier results were validated through bootstrap. The number of principal components (PC) in the experiments is indicated in parentheses

**Table 6:** Classification accuracies for the independent test set.

LTE, FE method:PCA					
**A2, NO(2PC)**	**A2, ME, NO(3PC)**	**A2, GL, NO(3PC)**	**A2, MM, NO(3PC)**	**A2, AG, NO(4PC)**	**A2, AG, MM(4PC)**

Total:92.3%(12/13)	Total:82.6%(19/23)	Total:65.1%(28/43)	Total:81.3%(13/16)	Total:64.2%(34/53)	Total:67.9%(36/53)
A2:100%(10/10)	A2:100%(10/10)	A2:90%(9/10)	A2:80.0%(8/10)	A2:90%(9/10)	A2:80.0%(8/10)
NO:66.7%(2/3)	ME:70.0%(7/10)	GL:53.3%(16/30)	MM:66.7%(2/3)	AG:57.5%(23/40)	AG:62.5%(25/40)
	NO:66.7%(2/3)	NO:100%(3/3)	NO:100%(3/3)	NO:66.7%(2/3)	MM:100%(3/3)
BER:0.17	BER:0.21	BER:0.19	BER:0.18	BER:0.29	BER:0.19

**LTE, FE method:Convex-NMF**					

**A2, NO(2SS)**	**A2, ME, NO(3SS)**	**A2, GL, NO(3SS)**	**A2, MM, NO(3SS)**	**A2, AG, NO(4SS)**	**A2, AG, MM(4SS)**

Total:92.3%(12/13)	Total:82.6%(19/23)	Total:67.4%(29/43)	Total:68.8(11/16)	Total:71.7%(38/53)	Total:64.2%(34/53)
A2:90.0%(9/10)	A2:90.0%(9/10)	A2:70.0%(7/10)	A2:50.0%(5/10)	A2:70.0%(7/10)	A2:60%(6/10)
NO:100%(3/3)	ME:70.0%(7/10)	GL:63.3%(19/30)	MM:100%(3/3)	AG:70.0%(28/40)	AG:62.5%(25/40)
	NO:100%(3/3)	NO:100%(3/3)	NO:100%(3/3)	NO:100%(3/3)	MM:100%(3/3)
BER:0.05	BER:0.13	BER:0.22	BER:0.17	BER:0.20	BER:0.26

**STE, FE method:PCA**					

**A2, NO(2PC)**	**A2, ME, NO(3PC)**	**A2, GL, NO(3PC)**	**A2, MM, NO(3PC)**	**A2, AG, NO(4PC)**	**A2, AG, MM(4PC)**

Total:92.3%(12/13)	Total:73.9%(17/23)	Total:76.7%(33/43)	Total:75.0(12/16)	Total:83.0%(44/53)	Total:73.6%(39/53)
A2:90.0%(9/10)	A2:80.0%(8/10)	A2:60.0%(6/10)	A2:60.0%(6/10)	A2:80.0%(8/10)	A2:70.0%(7/10)
NO:100%(3/3)	ME:70.0%(7/10)	GL:80.0%(24/30)	MM:100%(3/3)	AG:87.5%(35/40)	AG:72.5%(29/40)
	NO:66.7%(2/3)	NO:100%(3/3)	NO:100%(3/3)	NO:33.3%(1/3)	MM:100%(3/3)
BER:0.05	BER:0.28	BER:0.20	BER:0.13	BER:0.33	BER:0.19

**STE, FE method:Convex-NMF**					

**A2, NO(2SS)**	**A2, ME, NO(3SS)**	**A2, GL, NO(3SS)**	**A2, MM,NO(3SS)**	**A2, AG, NO(4SS)**	**A2, AG, MM(4SS)**

Total:92.3%(12/13)	Total:91.3%(21/23)	Total:90.7%(39/43)	Total:87.5(14/16)	Total:90.6%(48/53)	Total:83.0%(44/53)
A2:90.0%(9/10)	A2:90.0%(9/10)	A2:90.0%(9/10)	A2:80.0%(8/10)	A2:90.0%(9/10)	A2:90.0%(9/10)
NO:100%(3/3)	ME:90.0%(9/10)	GL:90.0%(27/30)	MM:100%(3/3)	AG:90.0%(36/40)	AG:80.0%(32/40)
	NO:100%(3/3)	NO:100%(3/3)	NO:100%(3/3)	NO:100%(3/3)	MM:100%(3/3)
BER:0.05	BER:0.07	BER:0.07	BER:0.07	BER:0.07	BER:0.10

Classification accuracies (total and by tumour type) and balanced error rates (BER) for the independent test set, using all the classification settings from Tables 4 and 5, for data at LTE and STE

### Determining the most adequate number of sources

One of the issues to which attention should be paid is the determination of the most appropriate number of sources for each problem. For this, we investigate the effect of varying the number of extracted sources on the classification results. For illustration, results for only one of the six previously investigated problems, namely A2, AG, MM, are presented. This problem is the most complex of those studied since it encompasses tumour type and grade, as well as extra or intra-axial origin discrimination: low grade neuroepithelial *vs*. high grade neuroepithelial, plus metastasis *vs*. low grade meningeal.

**Figure 4:**
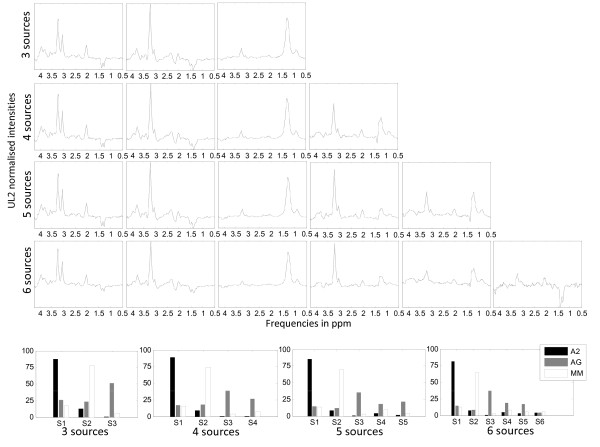
**Problem A2, AG, MM at LTE, varying the number of sources calculated**. Sources of the problem A2, AG, MM at LTE in different experiments with varying number of extracted sources. The first 4 rows show the sources corresponding to experiments in which 3, 4, 5 and 6 sources were calculated. Horizontal axis in the first four rows: frequency in ppm scale. The last row shows the percentage of contribution of each source to each tumour type, for each experiment. Horizontal axis in the last row: source signals. Vertical axes labels and representation of the sources as in previous figures.

**Figure 5:**
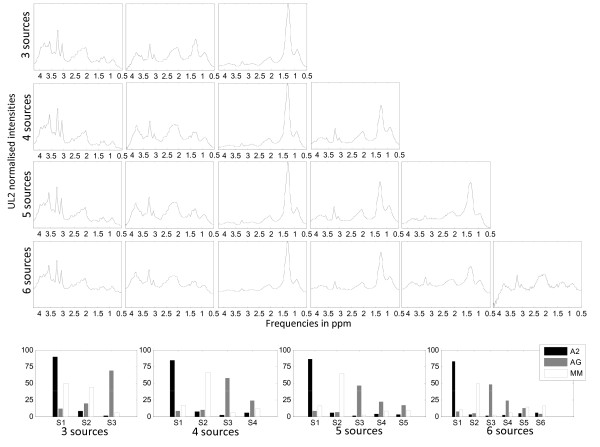
**Problem A2, AG, MM at STE, varying the number of sources calculated**. Sources of the problem A2, AG, MM at STE in different experiments with varying number of extracted sources. The first 4 rows show the sources corresponding to experiments in which 3, 4, 5 and 6 sources were calculated. Horizontal axis in the first four rows: frequency in ppm scale. The last row shows the percentage of contribution of each source to each tumour type, for each experiment. Horizontal axis in the last row: source signals. Axes labels and representation as in previous figures.

**Figure 6:**
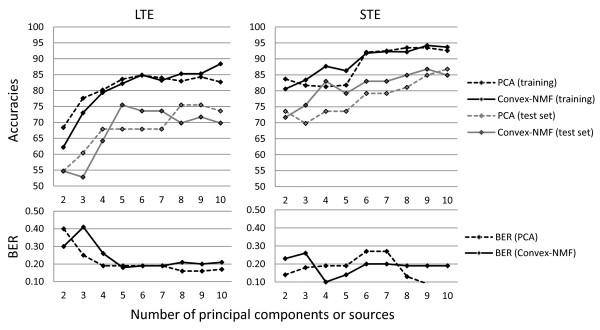
**Classification results for the problem A2, AG, MM at LTE and STE**. Plot for the comparison of the classification results for the problem A2, AG, MM at both LTE and STE, when using either PCA or Convex-NMF for DR, previous to classification with Fisher LDA. The left-hand side column corresponds to LTE results, and the right-hand side column to STE results. The first row displays the accuracy of the classification for all the methods, for training and test data sets. The second row displays the balanced error rate (BER) estimates for the test data sets. Horizontal axis: number of principal components or source signals. Vertical axis: accuracy and BER, respectively.

Figures 4 and 5 show the different sources obtained, at LTE and STE, respectively, when we vary the number of sources. The first four rows show the results of extracting 3, 4, 5 and 6 sources, while the last rows show the percentage of contribution of each source to each tumour type, for each experiment. Tables 7 and 8 compile the classification results when varying the number of sources from 2 to 10, for the training and the independent test set, respectively; and the plots in Figure 6 summarise the results, at LTE and STE.

**Table 7:** Classification results of A2, AG, MM for the training set, varying the number of extracted features.

PC/SS	LTE. PCA	LTE. Convex-NMF	STE. PCA	STE. Convex-NMF
2	Total:68.4% ± 3.4	Total:62.2% ± 3.6	Total:83.7% ± 2.6	Total:80.6% ± 2.7
	A2:75.0% ± 9.8	A2:55.4% ± 11.7	A2:86.8% ± 7.6	A2:78.0% ± 8.9
	AG:65.9% ± 4.1	AG:71.6% ± 4.2	AG:82.9% ± 3.3	AG:84.0% ± 3.3
	MM:71.0% ± 6.1	MM:45.7% ± 7.0	MM:84.3% ± 4.9	MM:74.3% ± 5.7

3	Total:77.6% ± 3.0	Total:73.0% ± 3.2	Total:81.7% ± 2.8	Total:83.4% ± 2.6
	A2: 95.0% ± 4.8	A2:90.2% ± 6.8	A2:85.7% ± 7.6	A2:95.4% ± 4.4
	AG: 71.5% ± 4.3	AG:63.6% ± 4.6	AG:81.3% ± 3.5	AG:81.7% ± 3.6
	MM: 83.5% ± 4.9	MM:85.4% ± 5.0	MM:80.8% ± 5.3	MM:82.7% ± 4.9

4	Total:80.2% ± 2.9	Total:79.4% ± 3.0	Total:81.3% ± 2.7	Total:87.7% ± 2.3
	A2:100% ± 0.0	A2:94.9% ± 5.2	A2:90.7% ± 6.4	A2:95.5% ± 4.6
	AG:75.9% ± 4.2	AG:72.5% ± 4.3	AG:80.6% ± 3.5	AG:86.3% ± 3.2
	MM:81.6% ± 5.2	MM:87.5% ± 4.3	MM:79.2% ± 5.3	MM:87.7% ± 4.3

5	Total:83.6% ± 2.7	Total:82.2% ± 2.9	Total:81.8% ± 2.7	Total:86.3% ± 2.4
	A2:100% ± 0.0	A2:100% ± 0.0	A2:90.8% ± 6.3	A2:90.6% ± 6.4
	AG:79.7% ± 3.9	AG:80.0% ± 3.9	AG:80.5% ± 3.6	AG:86.3% ± 3.1
	MM:85.5% ± 4.6	MM:80.3% ± 5.4	MM:81.2% ± 5.3	MM:84.6% ± 4.6

6	Total:84.8% ± 2.5	Total:84.9% ± 2.6	Total:92.1% ± 1.9	Total:91.8% ± 1.9
	A2:100% ± 0.0	A2:100% ± 0.0	A2:95.3% ± 4.6	A2:95.7% ± 4.1
	AG:81.7% ± 3.5	AG:82.8% ± 3.6	AG:92.7% ± 2.3	AG:91.2% ± 2.6
	MM:85.4% ± 4.8	MM:83.7% ± 4.9	MM:89.7% ± 4.1	MM:91.4 ± 3.8

7	Total:84.0% ± 2.7	Total:83.2% ± 2.7	Total:92.5% ± 1.9	Total:92.3% ± 1.9
	A2:100% ± 0.0	A2:100% ± 0.0	A2:95.6% ± 4.4	A2:91.2% ± 6.1
	AG:80.6% ± 3.8	AG:79.0% ± 4.0	AG:92.6% ± 2.3	AG:92.1% ± 2.4
	MM:85.2% ± 4.8	MM:85.7% ± 4.6	MM:91.2% ± 3.9	MM:93.0% ± 3.4

8	Total:83.0% ± 2.7	Total:85.3% ± 2.7	Total:93.5% ± 1.7	Total:92.2% ± 1.9
	A2:100% ± 0.0	A2:100% ± 0.0	A2:95.3% ± 4.6	A2:95.6% ± 4.5
	AG:78.7% ± 3.8	AG:80.7% ± 3.8	AG:93.0% ± 2.2	AG:91.2% ± 2.5
	MM:85.4% ± 4.9	MM:89.0% ± 4.3	MM:92.9% ± 3.4	MM:93.2% ± 3.3

9	Total:84.3% ± 2.6	Total:85.3% ± 2.6	Total:93.5% ± 1.7	Total:94.2% ± 1.7
	A2:100% ± 0.0	A2:100% ± 0.0	A2:95.3% ± 4.6	A2:95.5% ± 4.6
	AG:80.7% ± 3.6	AG:82.6% ± 3.5	AG:93.5% ± 2.2	AG:95.2% ± 1.9
	MM:85.5% ± 4.7	MM:85.5% ± 4.8	MM:92.9% ± 3.4	MM:91.4% ± 3.7

10	Total:82.7% ± 2.8	Total:88.4% ± 2.3	Total:92.6% ± 1.9	Total:93.7% ± 1.7
	A2:100% ± 0.0	A2:100% ± 0.0	A2:95.5% ± 4.5	A2:95.7% ± 4.5
	AG:79.1% ± 3.9	AG:87.0% ± 3.2	AG:91.9% ± 2.5	AG:93.5% ± 2.2
	MM:83.6% ± 4.9	MM:87.1% ± 4.5	MM:93.1% ± 3.4	MM:93.1% ± 3.3

Classification results (accuracy ± standard deviation) for the training set, at LTE and STE, obtained when varying the number of extracted features (principal components -PC- and source signals -SS-) from 4 to 10, for the problem A2, AG, MM. Fisher LDA was the classification method, and results were validated through bootstrap. The second and fourth columns show the results for PCA, and the third and fifth columns the results for Convex-NMF

**Table 8:** Classification results of A2, AG, MM for the independent test set, varying the number of extracted features.

PC/SS	LTE. PCA	LTE. Convex-NMF	STE. PCA	STE. Convex-NMF
2	Total:54.7%(29/53)	Total:54.7%(29/53)	Total:73.6%(39/53)	Total:71.7%(38/53)
	A2:60.0%(6/10)	A2:60.0%(6/10)	A2:90.0%(9/10)	A2:60.0%(6/10)
	AG:52.5% (21/40)	AG:50.0%(20/40)	AG:67.5%(27/40)	AG:72.5% (29/40)
	MM:66.7% (2/3)	MM:100% (3/3)	MM:100% (3/3)	MM:100% (3/3)
	
	BER:0.40	BER:0.30	BER:0.14	BER:0.23

3	Total: 60.4%(32/53)	Total:52.8%(28/53)	Total:69.8%(37/53)	Total:75.5%(40/53)
	A2:70.0%(7/10)	A2:60.0%(6/10)	A2:80.0%(8/10)	A2:80.0%(8/10)
	AG:55.0% (22/40)	AG:50.0%(20/40)	AG:65.0%(26/40)	AG:75.0%(30/40)
	MM:100% (3/3)	MM:66.7% (2/3)	MM:100% (3/3)	MM:66.7% (2/3)
	
	BER:0.25	BER:0.41	BER:0.18	BER:0.26

4	Total:67.9%(36/53)	Total:64.2%(34/53)	Total:73.6%(39/53)	Total:83.0%(44/53)
	A2:80%(8/10)	A2:60.0%(6/10)	A2:70.0%(7/10)	A2:90.0%(9/10)
	AG:62.5%(25/40)	AG:62.5%(25/40)	AG:72.5%(29/40)	AG:80%(32/40)
	MM:100%(3/3)	MM:100%(3/3)	MM:100%(3/3)	MM:100%(3/3)
	
	BER:0.19	BER:0.26	BER:0.19	BER:0.10

5	Total:67.9%(36/53)	Total:75.5%(40/53)	Total:73.6%(39/53)	Total:79.2%(42/53)
	A2:80%(8/10)	A2:70.0%(7/10)	A2:70.0%(7/10)	A2:80.0%(8/10)
	AG:62.5%(25/40)	AG:75.0%(30/40)	AG:72.5%(29/40)	AG:77.5%(31/40)
	MM:100%(3/3)	MM:100%(3/3)	MM:100%(3/3)	MM:100%(3/3)
	
	BER:0.19	BER:0.18	BER:0.19	BER:0.14

6	Total:67.9%(36/53)	Total:73.6%(39/53)	Total:79.2%(42/53)	Total:83.0%(44/53)
	A2:80%(8/10)	A2:70.0%(7/10)	A2:70.0%(7/10)	A2:90.0%(9/10)
	AG:62.5%(25/40)	AG:72.5%(29/40)	AG:82.5%(33/40)	AG:82.5%(33/40)
	MM:100%(3/3)	MM:100%(3/3)	MM:66.7%(2/3)	MM:66.7%(2/3)
	
	BER:0.19	BER:0.19	BER:0.27	BER:0.20

7	Total:67.9%(36/53)	Total:73.6%(39/53)	Total:79.2%(42/53)	Total:83.0%(44/53)
	A2:80%(8/10)	A2:70.0%(7/10)	A2:70.0%(7/10)	A2:90.0%(9/10)
	AG:62.5%(25/40)	AG:72.5%(29/40)	AG:82.5%(33/40)	AG:82.5%(33/40)
	MM:100%(3/3)	MM:100%(3/3)	MM:66.7%(2/3)	MM:66.7%(2/3)
	
	BER:0.19	BER:0.19	BER:0.27	BER:0.20

8	Total:75.5%(40/53)	Total:69.8%(37/53)	Total:81.1%(43/53)	Total:84.9%(45/53)
	A2:80%(8/10)	A2:70.0%(7/10)	A2:80%(8/10)	A2:90.0%(9/10)
	AG:72.5%(29/40)	AG:67.5%(27/40)	AG:80%(32/40)	AG:85%(34/40)
	MM:100%(3/3)	MM:100%(3/3)	MM:100%(3/3)	MM:66.7%(2/3)
	
	BER:0.16	BER:0.21	BER:0.13	BER:0.19

9	Total:75.5%(40/53)	Total:71.7%(38/53)	Total:84.9%(45/53)	Total:86.8%(46/53)
	A2:80%(8/10)	A2:70.0%(7/10)	A2:90%(9/10)	A2:90%(9/10)
	AG:72.5%(29/40)	AG:70.0%(28/40)	AG:82.5%(33/40)	AG:87.5%(35/40)
	MM:100%(3/3)	MM:100%(3/3)	MM:100%(3/3)	MM:66.7%(2/3)
	
	BER:0.16	BER:0.20	BER:0.09	BER:0.19

10	Total:73.6%(39/53)	Total:69.8%(37/53)	Total:86.8%(46/53)	Total:84.9%(45/53)
	A2:80%(8/10)	A2:70.0%(7/10)	A2:90%(9/10)	A2:90.0%(9/10)
	AG:70%(28/40)	AG:67.5%(27/40)	AG:85%(34/40)	AG:85%(34/40)
	MM:100%(3/3)	MM:100%(3/3)	MM:100%(3/3)	MM:66.7%(2/3)
	
	BER:0.17	BER:0.21	BER:0.08	BER:0.19

Classification accuracies (total and by tumour type) and BER for the independent test set, at LTE and STE, using the corresponding classification settings from Table 7. The second and fourth columns show the results for PCA, and the third and fifth columns the results for Convex-NMF

## Discussion

### NMF as a source extraction method

The results reported in Tables 1 and 2 clearly indicate that, in terms of correlation, the Convex-NMF method consistently outperforms the rest, yielding better results in nearly every experiment. The advantage of Convex-NMF is especially striking at STE (results in Table 2). Regarding the different initialisation alternatives, correlation results do not show much dependence on the type of initialisation strategy. *Random *and *K-means*-based initialisations seem to be, overall, the best choices at both times of echo. Therefore, in all subsequent analyses, Convex-NMF with K-means initialisation was the selected method.

The illustrative example of Figure 1, in which the NMF-extracted sources are shown, reveals an effect resulting from the fact that the best correlation value between the mean of the spectra of a class and the sources is used as the indicator for selecting the source that best represents that class: for some types of NMF, this approach results in situations in which each source does not necessarily correspond univocally to a single class; sometimes, instead, a single source may encompass more than one class. This can be clearly seen in Figure 1, where the sources calculated with the first four methods (*euc, als, alspg*, and *alsobs*) can be explained as follows: the ones in the leftmost column describe mostly the A2 and MM tumour types, respectively (the correlation values can be seen in Table 1); the ones in the rightmost column describe the normal tissue; and the ones in the middle column have a low correlation with the three tumour types in the experiment, as evidenced if we compare them qualitatively to the mean spectra at the bottom of the Figure.

In stark contrast, we can also conclude from Figure 1 (and from further results not reported here, but which are consistent with the high correlation values shown in Tables 1 and 2) that Convex-NMF performs class-specific source extraction far better than the other methods studied. It is remarkable how Convex-NMF is able to extract sources that represent each class univocally. Here, A2 is represented by one source (leftmost column in Figure 1), meningiomas by another source (middle column of Figure 1) and normal tissue by a third one (rightmost column in Figure 1). This way, Convex-NMF extremely simplifies the interpretation of the source signals extracted. For example, while the sources produced by the *euc, als, alspg*and *alsobs *methods show a doublet at about 1.5 ppm (Alanine), the two sources for A2 and MM in the *convex *method clearly discriminate the contribution from the Lactate inverted doublet centred at 1.35 ppm, typical of A2, from the Alanine inverted doublet centred at 1.45 ppm, which is typical from meningioma.

### Labelling using convex-NMF

The results reported in Table 3, and Figures 2 and 3 show that normal brain (NO) is perfectly discriminated in all of the comparisons carried out, as it might be expected due to the metabolic differences of healthy tissue with respect to brain tumours in general. Furthermore, the differential discrimination among meningeal, glial (A2) and control is reasonably good for both times of echo (89-97%). On the other hand, the trilateral discrimination between the aggressive tumours (ME or GL), A2 and NO is far less accurate, reaching a low 71% for the A2, GL, NO at LTE. The detailed interpretation of the last two diagnostic problems, involving the aggressive grade IV superclass is as follows:

• Problem A2 *vs*. AG *vs*. NO at LTE: A2 is fully represented by one of the sources (Figure 2, first row, column S1), which correlates at 0.98 with the mean spectrum of A2; AG is labelled with an accuracy of 70.6% and it is mostly represented by two sources (Figure 2, first row, columns S3 and S4), which correlate at 0.97 and 0.67 with the mean spectrum of AG; finally, NO is also fully represented by one of the sources (Figure 2, first row, column S2), which fully correlates (1.0) with the mean spectrum of NO. The accuracy for the groups A2 and NO is 100%, while it falls to 70% for AG, which totals 77.8% of correctly labelled samples.

• Problem A2 *vs*. AG *vs*. NO at STE: A2 is labelled with an accuracy of 81.8% and it is represented almost exclusively by one of the sources (Figure 2, second row, column S1), which correlates at 0.99 with the mean spectrum of A2;AG is labelled with an accuracy of 92.7% and it is mostly represented by two sources (Figure 2, second row, columns S3 and S4), which correlate at 0.94 and 0.98 with the mean spectrum of AG; and NO is fully represented by one of the sources (Figure 2, second row, column S2), which fully correlates (1.0) with the mean spectrum of NO. At STE, the highest accuracy for AG raises the overall accuracy to 92.3%. The higher accuracy for short echo time classifiers is also common in other studies based in supervised analysis of data (i.e. [1]).

• Problem A2 *vs*. AG *vs*. MM at LTE: A2 is labelled with an accuracy of 95% and it is represented almost exclusively by one of the sources (Figure 3, first row, column S1), which correlates at 0.99 with the mean spectrum of A2; AG is labelled with an accuracy of 64.2% and it is mostly represented by two sources (Figure 3, first row, columns S3 and S4), which correlate at 0.96 and 0.66 with the mean spectrum of AG; finally MM is labelled with an accuracy of 85.5

• Problem A2 *vs*. AG *vs*. MM at STE: A2 is labelled with an accuracy of 90.9% and it is represented almost exclusively by one of the sources (Figure 3, second row, column S1), which correlates at 0.98 with the mean spectrum of A2; AG is labelled with an accuracy of 85.5% and it is mostly represented by two sources (Figure 3, second row, columns S3 and S4), which correlate at 0.94 and 0.93 with the mean spectrum of AG; finally MM is labelled with an accuracy of 86.2% and represented almost in full by one of the sources (Figure 3, second row, column S2), which correlates at 0.97 with the mean spectrum of MM. The overall accuracy is 86.3%. Again, at STE the accuracy for AG is higher than at LTE.

The results for the AG superclass illustrate that Convex-NMF is not always successful in extracting tumour type-specific sources. Two inherent characteristics of AG may explain this: first, AG has been artificially built using two tumour types (ME andGL) and, second, GL by itself is a rather heterogeneous type in which plenty of substructure can be found [35,40-43].

This does not preclude the interpretation of the sources. According to the signal profile and its metabolic interpretation, one of the sources representing AG (Figure 2, first row, column S3) seems to correspond to the necrotic core (high mobile lipids, ML) [34,44]; while the other (Figure 2, first row, column S4) seems to correspond to the cellular part of the tumour (high total choline, indicating high proliferation rate [45]). Note that this dichotomy is valid for both echo times, and the two problems above, in which one source represents the cellular part while the other represents the necrotic core, and both are needed to accurately recognise SV patterns of GL or ME.

### Convex-NMF as DR method prior to classification

The comparison of the results of Tables 4 and 5 reveals that at STE the classification results for the training dataset improve in all the experiments when using Convex-NMF for feature extraction instead of PCA, prior to standard supervised classification. This pattern was repeated for LTE, with the exception of the A2, AG, MM problem, which yielded a poorer result; in any case, the difference is rather small and not significant. Interestingly, the unsupervised labelling results reported in Table 3, though worse than those of their supervised counterparts reported in Table 4, are still comparable to those obtained with PCA and LDA in fully supervised mode (in fact, they are consistently better for STE, while worse for LTE).

An independent test set was then used to further validate the robustness of the developed classifiers for data preprocessed with both FE methods: PCA and the orthogonal Convex-NMF sources. Table 6 contains the accuracy results (total and by tumour type), as well as the corresponding balanced error rate (BER) [9]. Again, at STE, the use of Convex-NMF orthogonal sources yields results that clearly outperform those of PCA-based classification. However, at LTE the results are more mixed: similar in the cases of A2, NO; A2, ME, NO and A2, GL, NO; better in the case of A2, AG, NO; and worse in remaining two: A2, AG, MM (with a small difference) and A2, MM, NO, with a more noticeable difference.

Other studies have addressed similar problems in the existing literature, for similar data. We report next some of these results for comparative purposes, although the techniques and the evaluation criteria involved are not always the same.

• In [7], as first step of a multiclass classifier for data acquired at LTE, aggressive tumours (AG) were discriminated from A2 with an accuracy of 84.7%. In our experiments, which also include the healthy tissue class, an 85.1% accuracy was achieved from the extracted sources. For the same problem, with data acquired at STE, an accuracy of 90.9% was reported in [38], to be compared with a 92.3% obtained in our study from the sources.

• In [1], when classifying low-grade meningiomas (MM) vs. low-grade glial tumours (A2, plus oligodendrogliomas and oligoastrocytomas, two tumour types not analysed in our experiments) vs. high-grade aggressive tumours (AG), the reported accuracies for the training set were 84.2% at LTE and 89.0% at STE, while the accuracies for an independent test set were, in turn, 69.8% and 82.5%. The results obtained in our experiments when separating A2 from AG and MM, using the sources, were 79.4% at LTE and 87.7% at STE, for training; and 64.2% at LTE and 83.0% at STE, for the independent test set.

### Determining the most adequate number of sources

Figures 4 and 5 show the different sources obtained, at LTE and STE, respectively, when varying the number of sources. In both figures, the first columns of sources are representing mostly the A2 type; the second columns represent mostly the MM type; and, finally, the third columns are mainly representing necrotic tissue, which should only be found in GL and ME. It is interesting to see how, when calculating 4 sources, the first 3 sources remain, while the new one seems to correspond to actively proliferating tumour (high total choline at ca. 3.21 ppm).

The bar plots for 4 sources, at both times of echo, show the extent to which sources 3 (necrotic tissue) and 4 (proliferative tumour) are representing the AG superclass. At LTE, when calculating 5 sources, the first four look very similar to those calculated in the experiment with 4 sources, while the new one seems to express part of the AG superclass, which is now in fact split into the last three sources. The non-necrotic 4th and 5th sources would show an inverted trend for total choline (ca. 3.21 ppm) versus ML/Lactate (ca. 1.3 ppm). Then, decreasing choline would be matched by increased ML/Lactate, suggesting sampling of aggressive tumour subtypes with variable proliferation rate (total Choline), with concomitant effects on the lactate and ML accumulation. At STE, when calculating 5 sources, the first four also look very similar to those obtained in the experiment with only 4 sources, but the new one is not only part of AG, but also partly of MM.

Six sources at LTE already seem to be too many, given that the contribution of the last one is comparatively very small and completely unspecific. Six sources at STE also seem to be too many. In this case, the MM class is less represented by the second source, while the 5th does contribute both to AG and MM. This could have contributions from class outlier cases (atypical meningiomas), for which mobile lipids could be starting to increase. The last one could contain some artefactual bad water suppression above 3.7 ppm. Up to this point, and based solely on the patterns of the sources, and the percentages of contribution of these to each class, choosing 4 or 5 sources seems to be best option, at both times of echo, to maintain the correspondence between source, or set of sources, and individual tumour types.

Tables 7 and 8 compile the classification results corresponding to the varying number of sources (from 2 to 10), both for the training and the independent test set, respectively. The plots in Figure 6 summarise these results. The leftmost column in this figure contains the results at LTE, and the rightmost column, the results at STE. Strictly in terms of classification, the use of 5 sources seems to be a good choice at LTE, given the accurate results obtained with the independent test set, and its low BER value. At STE, choosing 4 sources seems to be a good compromise, for which the accuracies for the training and the independent test set are high, while the BER for the test set stays the lowest.

## Conclusions

The unsupervised analysis of SV ^1^H-MRS data from human brain tumours using Convex-NMF has been shown to produce a reduced number of sources that can be confidently recognised as representing brain tumour types or healthy tissue in a way that other source extraction methods, including other NMF variants, cannot. Importantly, this result allows us to produce class assignments for unlabelled spectra in fully unsupervised mode, using the mixing matrix directly as a basis for classification, with results that are comparable to those obtained in fully supervised mode. The use of the sources extracted by Convex-NMF for dimensionality reduction leads to simple LDA-based classifiers with independent test performances that are comparable with, and are often better than previously described strategies. In summary, the unsupervised properties of Convex-NMF place this approach one step ahead of classical label-requiring supervised methods for detection of the increasingly recognised molecular subtype heterogeneity within human brain tumours. The application of Convex-NMF in computer assisted decision support systems is expected to facilitate further improvements in the uptake of MRS-derived information by clinicians.

## Authors' contributions

SOM, PJGL and CA conceived the overall scope of the study. MJS participated in the data processing. SOM implemented the methods and carried out the experiments. SOM, PJGL, and AV designed the set of experiments, and analysed the results from the machine learning viewpoint. MJS and CA contributed the biochemical and spectroscopic analysis of the results. PJGL, AV and CA coordinated the work. All authors helped to draft the manuscript and approved its final version.

## Endnote

^a ^http://gabrmn.uab.es/interpret

^b ^http://gabrmn.uab.es/sc
